# The possible existence of occult metastasis in patients with ovarian clear-cell carcinoma who underwent complete resection without any residual tumours

**DOI:** 10.18632/oncotarget.23921

**Published:** 2018-01-04

**Authors:** Hiroaki Kajiyama, Shiro Suzuki, Masato Yoshihara, Kimihiro Nishino, Nobuhisa Yoshikawa, Fumi Utsumi, Kaoru Niimi, Mika Mizuno, Michiyasu Kawai, Hidenori Oguchi, Kimio Mizuno, Osamu Yamamuro, Tetsuro Nagasaka, Kiyosumi Shibata, Fumitaka Kikkawa

**Affiliations:** ^1^Department of Obstetrics and Gynecology, Graduate School of Medicine, Nagoya University, Nagoya, Japan; ^2^ Department of Gynecology, Aichi Cancer Center Hospital, Nagoya, Japan; ^3^Department of Obstetrics and Gynecology, Toyohashi Municipal Hospital, Toyohashi, Japan; ^4^Department of Obstetrics and Gynecology, Toyota Memorial Hospital, Toyota, Japan; ^5^Department of Obstetrics and Gynecology, Nagoya First Red-Cross Hospital, Nagoya, Japan; ^6^Department of Obstetrics and Gynecology, Nagoya Second Red-Cross Hospital, Nagoya, Japan; ^7^Division of Medical Laboratory Sciences, School of Health Science, Nagoya University, Nagoya, Japan; ^8^Department of Obstetrics and Gynecology, Banbuntane Hotokukai Hospital, Fujita Health University, Nagoya, Japan

**Keywords:** epithelial ovarian carcinoma, clear-cell carcinoma, recurrence, metastasis

## Abstract

The objective of this study was to estimate the frequency of possible occult metastasis through long-term survival analyses in patients with clear cell carcinoma (CCC) who had undergone complete resection. During the period of 1990-2015, 799 patients with stage I-IV CCC were identified in the TOTSG database. Of these, a total of 528 patients without a residual tumor were enrolled in the study and classified into four groups: Group 1: FIGO stage IA-IB (N=104), Group 2: FIGO stage IC1 (N=170), Group 3: FIGO stage IC2/IC3 (N=98), and Group 4: FIGO stage II-III (no residual tumor: N=156). Cumulative incidences of recurrence (CIR) and death (CID) were examined. The median age was 54, ranging from 29-87. The 5-year CIR / CID of each group were as follows: Group 1 (7.3% / 3.8%), Group 2 (14.3% / 10.2%), Group 3 (37.7% / 18.4%), and Group 4 (46.5% / 33.8%), respectively {*P*<0.0001 (recurrence) / *P*<0.0001 (death)}. Furthermore, confining analysis to relapsed patients, 1-, 2-, and 3-year CID after recurrence were 41.5, 60.9, and 73.9, respectively. Confining analyses to patients with sufficient information about adjuvant chemotherapy, the 5-year CIR / CID of stage IA-IC1 patients with or without chemotherapy were as follows: recurrence {13.0% (yes) / 9.6% (no)}, death {9.3% (yes) / 4.2% (no)}, respectively {*P*=0.947 (CIR) / *P*=0.224 (CID)}. CCC patients staged greater than IC2/ IC3 show a marked risk of mortality, even after complete surgical resection.

## INTRODUCTION

Clear-cell carcinoma of the ovary (CCC) is a comparatively rare malignancy in Western countries, accounting for approximately less than 10% of all ovarian carcinomas [1]. However, this histological type is very common in East Asia; CCC is the second most frequent tumor of epithelial ovarian carcinoma in Japan, representing 20-25% [2]. Generally speaking, CCC displays a discriminative clinical behavior compared with other histological types of epithelial ovarian carcinomas. This tumor frequently demonstrates comparatively slow-growing characteristics, leading to presentation at earlier stages [3]. According to prior studies, CCC is known as an aggressive subtype of malignant ovarian neoplasm due to comparatively lower-level sensitivity to platinum-compound chemotherapy, which results in poorer oncologic outcomes of CCC patients [4].

Accordingly, the extent of cytoreductive surgery is one of the major prognostic determinants for patients with CCC. In fact, previous studies showed that only complete surgical resection without any macroscopic residual tumors (RT) could improve the prognosis of advanced CCC patients [5]. Thus, to date, gynecologic oncologists have made maximal efforts to achieve complete cytoreductive surgery. On the other hand, confining analysis to stage I tumors, CCC patients at stage IC2 and IC3 show a greater risk of recurrence and poorer survival than those with stage IA despite platinum-based adjuvant chemotherapy [6]. The possible rationale for this phenomenon is thought to be due to invisible occult metastasis throughout the body, including peritoneal cavity, node, and parenchymal organs. Although, needless to say, surgeons’ eagerness to perform complete resection is important, the evidence on long-term recurrence and mortality in patients without any macroscopic RT is insufficient. Furthermore, we should clarify to what extent the microscopic occult tumors influence the oncologic outcome of patients with CCC, even after successful complete resection.

To evaluate the oncologic outcome of patients with CCC without any macroscopic RT and to determine the impact of surgery, we conducted a retrospective study analyzing 528 patients who were accumulated in a total of 14 Japanese University / general hospitals and assessed based on the central pathological review system.

## RESULTS

### Patients’ characteristics

The characteristics of enrolled patients are presented in Table 1. The median (range) age was 54 (29–87 years) years. The median follow-up for surviving patients was 74.1 months. The distributions of the FIGO stage were 70.5% (372/528) for stage I, 15.3% (81/528) for stage II, and 14.2% (75/528) for stage III. The distributions of the stage I substages were as follows: IA: 102 (19.3%), IB: 2 (0.4%), IC1: 170 (32.2%), IC2: 51 (9.7%), and IC3: 47 (8.9%). The patient distributions by stratification based on the starting period of the initial treatment were: before 1999: 105 (19.9%), 2000-2004: 107 (20.3%), 2005-2009: 143 (27.1%), and that after 2010: 173 (32.8%). Eighty-one patients (15.3%) received conventional platinum-based chemotherapy, and 344 patients (65.2%) received taxane plus platinum chemotherapy. In 24 patients, detailed information on chemotherapy was lacking. In the majority of the patients (N=330: 62.5%), the preoperative CA125 was elevated to over 35 U/mL.

**Table 1:** Patients' characteristics

	N	%
Total	528	
Age		
(Median range)	54 (29-87)	
—39	50	9.5
40-49	118	22.3
50-59	225	42.6
60-69	99	18.8
70—	34	6.4
FIGO stage		
I	372	70.5
IA	102	19.3
IB	2	0.4
IC1	170	32.2
IC2	51	9.7
IC3	47	8.9
II	81	15.3
III	75	14.2
Period of initial treatment		
—1999	105	19.9
2000-2004	107	20.3
2005-2009	143	27.1
2010—	173	32.8
Chemotherapy		
Platinum-based	81	15.3
Taxane plus platinum	344	65.2
Others	11	2.1
None	68	12.9
Unknown	24	4.5
CA125 value		
≤ 35 U/mL	180	34.1
> 35 U/mL	330	62.5
Unknown	18	3.4

FIGO: International Federation of Gynecology and Obstetrics, IC substage was defined according to FIGO 2014 classification.

### Oncologic outcome

During the follow-up of the total of 528 patients, 142 patients (26.9%) developed recurrence. Confining analysis to those relapsed patients, the median time to recurrence was 15.5 months. In addition, 95 patients (18.0%) died of their recurrence. The CIR curves of CCC patients belonging to each stage is shown in Supplementary Figure 1.

**Figure 1:**
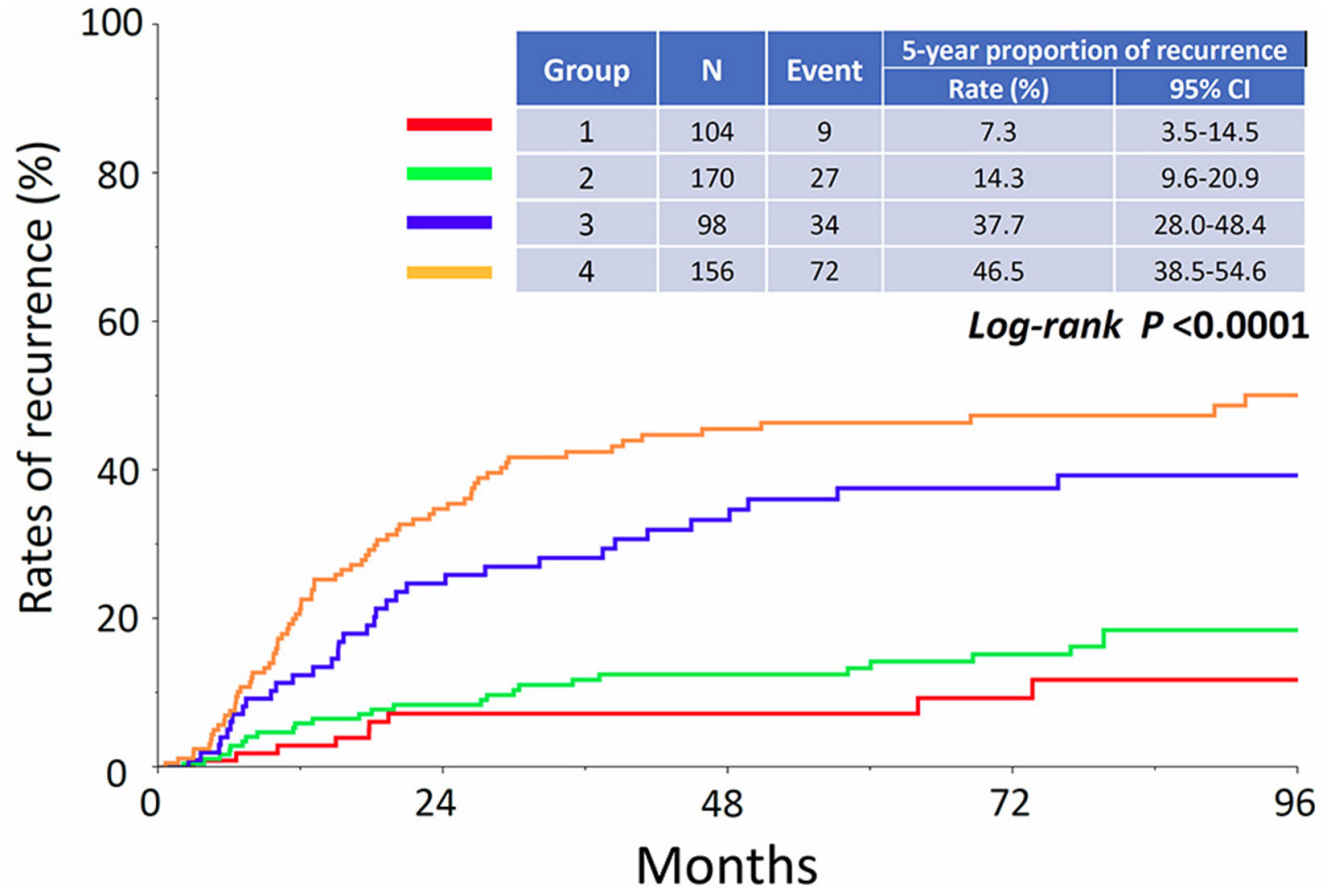
Cumulative incidence of recurrence in patients who belong to Groups 1, 2, 3, and 4

**Figure 2:**
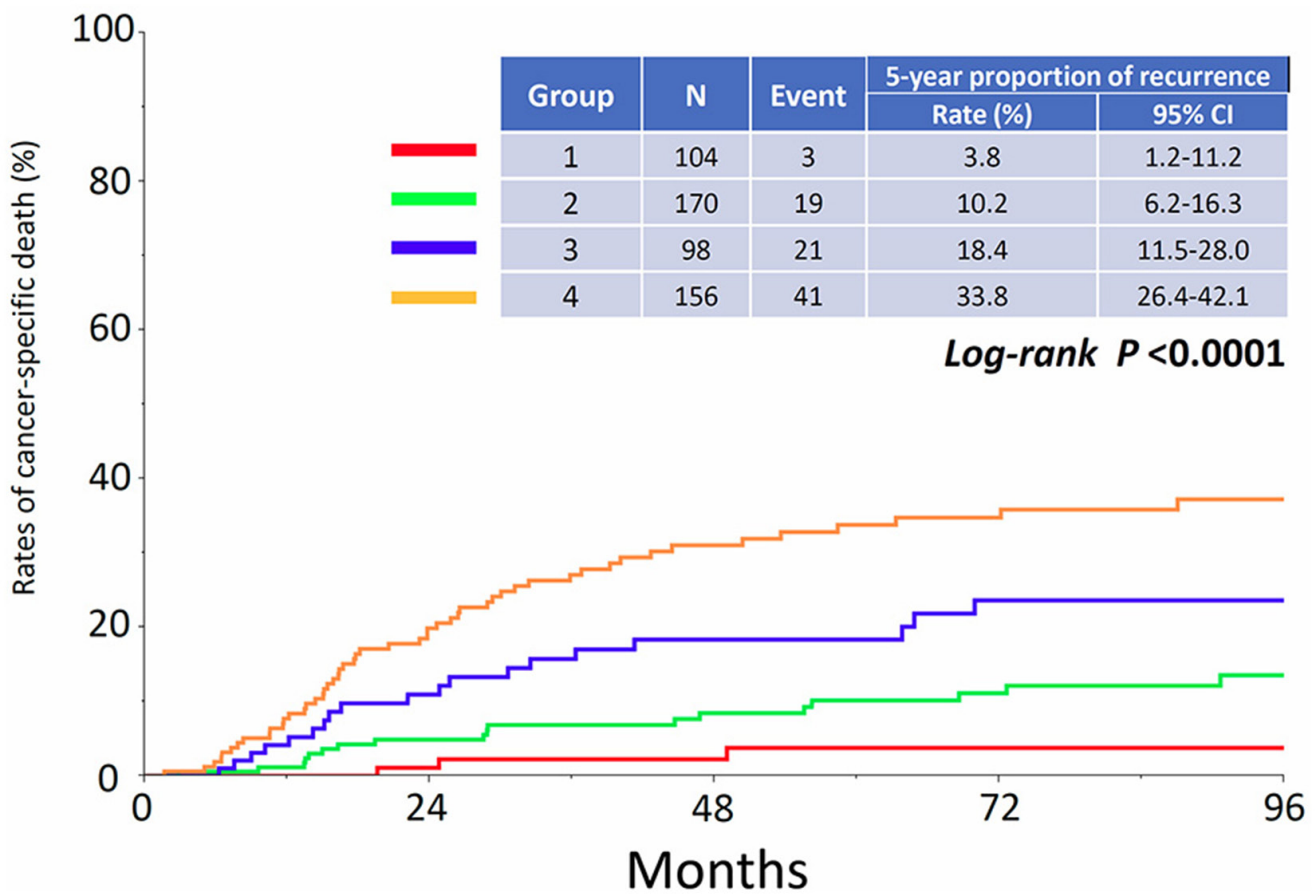
Cumulative incidence of cancer-specific death in patients who belong to Groups 1, 2, 3, and 4

**Figure 3:**
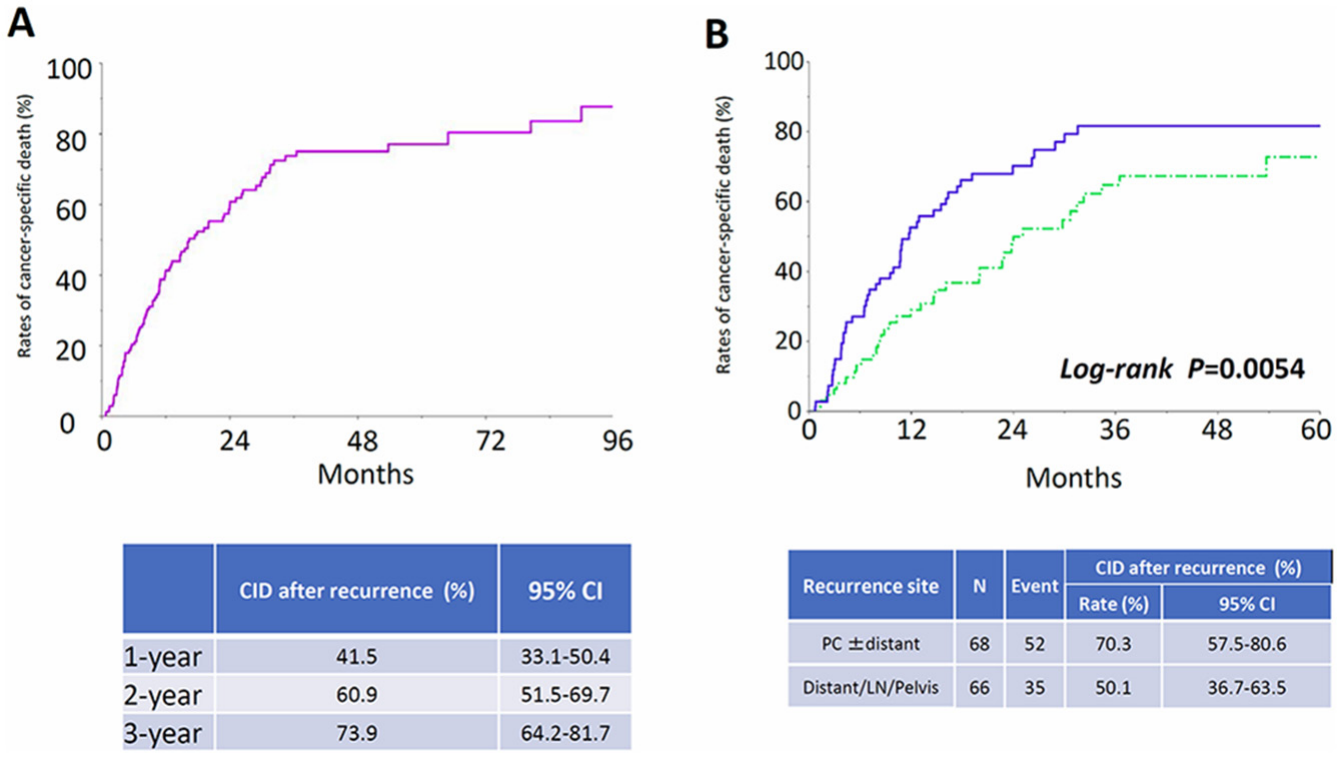
Cumulative incidence of cancer-specific death after recurrence in relapsed patients **(A)** All relapsed patients, **(B)** On stratification by recurrence site. Blue line: PC ± distant, Green line: Distant/LN/Pelvis.

Figure 1 shows the proportion of recurrence in each group. The 5-year CIR of patients in each group were as follows: Group 1: 7.3% (95% CI: 3.5-14.5), Group 2: 14.3% (95% CI: 9.6-20.9), Group 3: 37.7% (95% CI: 28.0-48.4), and Group 4: 46.5% (95% CI: 38.5-54.6), respectively (*P*<0.0001). In addition, the CID of patients in each group are listed in Figure 2. The 5-year CID were as follows: Group 1: 3.8% (95% CI: 1.2-11.2), Group 2: 10.2% (95% CI: 6.2-16.3), Group 3: 18.4% (95% CI: 11.5-28.0), and Group 4: 33.8% (95% CI: 26.4-42.1), respectively (*P*<0.0001). Statistical comparisons between each group regarding recurrence and death are demonstrated in Table 2. All comparisons except for the recurrence rates between Groups 1 and 2 were significant or marginally significant. Of notable importance, the poor prognosis of patients in Group 3 (stage IC2 /IC3) was closer to that of those in Group 4 (stage II-III), although there was no significant difference between the two groups. Furthermore, as we mentioned above, we identified 142 patients who developed recurrence. We examined the rate of cancer-specific death after recurrence in those patients. Figure 3A shows that the median postrecurrence survival time was 16.5 months. Confining analysis to those patients, the 1-, 2-, and 3-year CID after recurrence were 41.5% (95% CI: 33.1-50.4), 60.9% (95% CI: 51.5-69.7), and 73.9% (95% CI: 64.2-81.7), respectively. Figure 3B shows postrecurrence survival curves on stratification by recurrence site (P=0.0054).

**Table 2:** Significance of differences between groups^*^

	Proportion of recurrence				Proportion of death			
	Group 1	Group 2	Group 3	Group 4	Group 1	Group 2	Group 3	Group 4
**Group 1**	-	0.175	<0.0001	<0.0001	-	0.031	<0.0001	<0.0001
**Group 2**	-	-	<0.0001	<0.0001	-	-	0.013	<0.0001
**Group 3**	-	-	-	0.080	-	-	-	0.057
**Group 4**	-	-	-	-	-	-	-	-

^*^: Log-rank test

**Figure 4:**
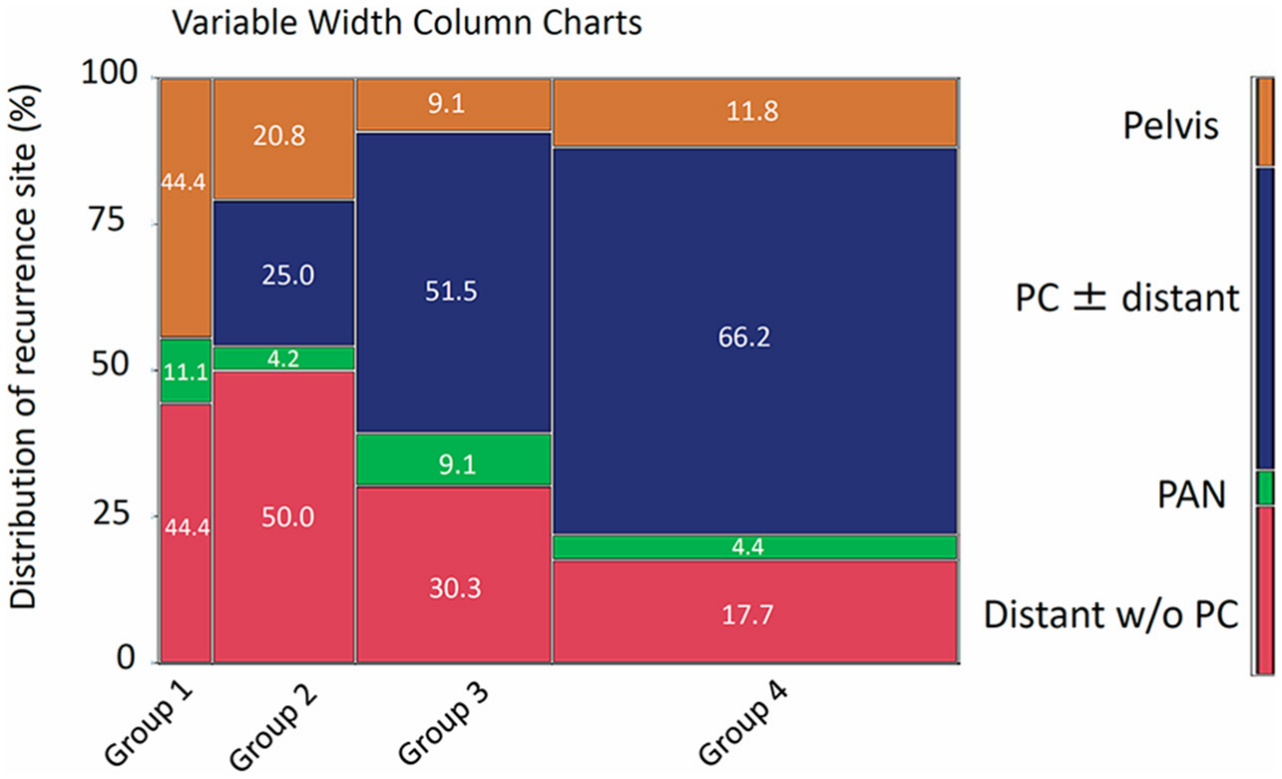
Distributions of recurrence site in patients who belong to each group (variable width column charts) PC: peritoneal cavity, PAN: paraaortic lymph node, distant: distant metastasis in parenchymal organ.

We subsequently examined the site of recurrence in patients who developed recurrence. Figure 4 shows distributions of recurrence sites in patients belonging to each group (variable width column charts). The rates of recurrence including the peritoneal cavity were 0, 25.0, 51.5, and 66.2% in patients belonging to Groups 1, 2, 3, and 4, respectively. Patients in the higher staged group showed a higher rate of recurrence in the peritoneal cavity (Cochran–Armitage test for trend, *P*<0.0001) (Figure 4).

**Figure 5:**
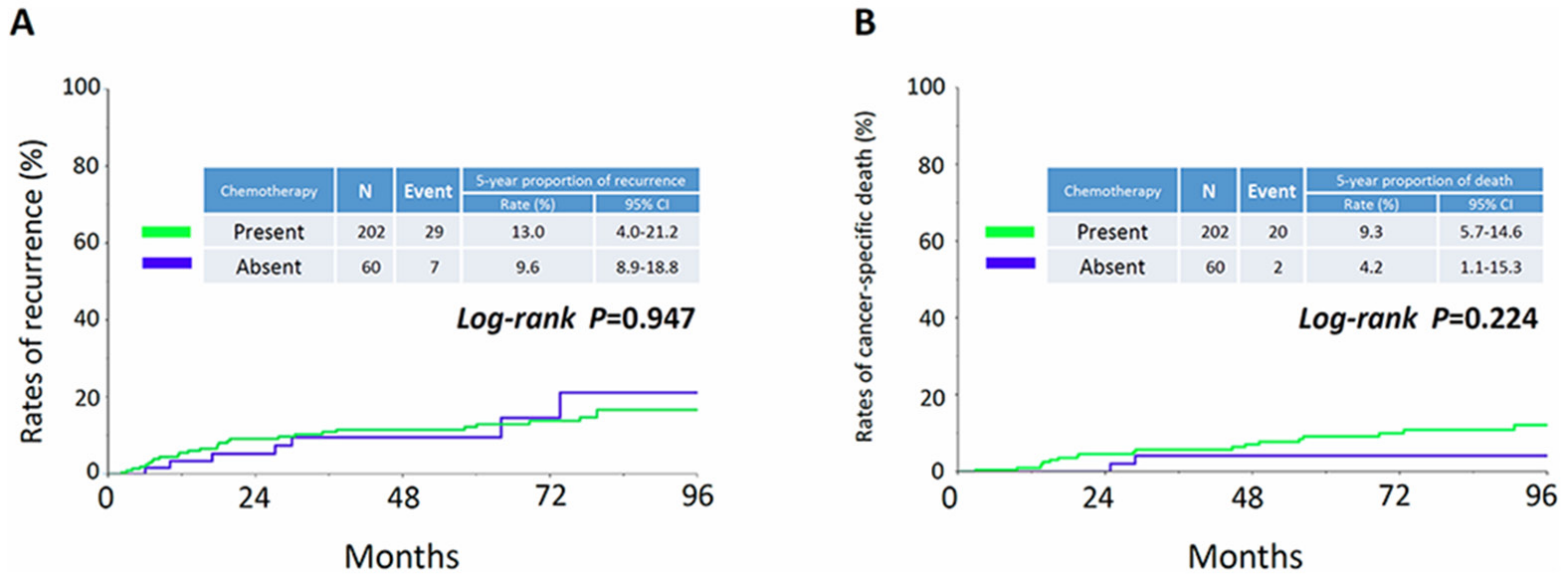
Cumulative incidence of recurrence **(A)** and death **(B)** by stratification to the presence or absence of adjuvant chemotherapy in stage IA-IC1 patients.

The above-mentioned results are likely to reflect the higher frequency of occult metastasis. The ability to reduce these invisible tumors may depend on the effect of postoperative chemotherapy. Indeed, in our study, we had clinical information on postoperative adjuvant chemotherapy in 504 (95.5%) patients. Thus, we finally examined whether stage IA-IC1 patients who underwent adjuvant chemotherapy showed a more favorable clinical outcome. Figure 5A-5B shows the CIR or CID by stratification to the presence or absence of adjuvant chemotherapy. We did not identify any significant differences in rates of recurrence or death between the two cohorts {5-year recurrence rate: 13.0% (present) vs. 9.6% (absent): *P*=0.947, mortality rate: 9.3% (present) vs. 4.2% (absent): *P*=0.224}.

## DISCUSSION

Recurrence essentially arises from “seeds” of an invisible tumor that was not successfully removed by various treatments or the body’s immune system. Thus, recurrence does not occur without occult metastases; we merely could not radiologically detect or recognize them at the end of a series of initial treatments. Actually, despite our efforts, it is difficult to macroscopically identify a one-millimeter-sized tumor behind intraperitoneal organs during surgery. Especially, prior studies reported the importance of the complete resection of CCC, reflecting the fact that this histological type is intrinsically chemoresistant [2, 5]. Indeed, the results of a large-scale retrospective study provided evidence that the residual tumor status is an absolute independent prognostic factor for CCC patients [5]. However, even if we achieve the complete surgical removal of all visible tumors in women with CCC, those patients will experience recurrence at a certain rate. These facts prompted us to question how much the recurrence and mortality rate of CCC patients differed according to the extent of the potentially remaining tumor. Thus, in the present study, we first classified a total of 528 CCC patients who underwent successful complete tumor resection into four groups: Group 1: FIGO stage IA-IB, Group 2: FIGO stage IC1, Group 3: FIGO stage IC2/IC3, and Group 4: FIGO stage II-III (no residual tumor). As we expected, a poorer prognosis was noted in patients who belonged to the higher group, probably reflecting an increased extent of invisible occult tumors. More importantly, a poor prognosis of patients in Group 3 (stage IC2 /IC3) was closer to that of those in Group 4 (stage II-III) than those in Group 2 (stage IC1). Certainly, to date, several studies have demonstrated that a capsule status with surface involvement, or positive cytology leads to an unfavorable outcome in CCC patients with a stage IC tumor (R). We re-realized that CCC patients with preoperative capsule rupture or positive ascites exhibited a marked recurrence / mortality risk, and they are considered as a different entity, although they belong to the same IC group. Therefore, reflecting on these different backgrounds of the oncologic outcome, stage IC2 /IC3 CCC may be recognized as a near-stage II-III tumor, instead of stage I, at least in actual clinical practice.

In the present study, 142 CCC patients eventually experienced recurrence. Confining analysis to those relapsed patients, the median time to recurrence was 15.5 months. Interestingly, the most common relapsed site was the peritoneal cavity regardless of other distant sites of parenchymal recurrence. Of note, a higher frequency of intraperitoneal recurrence was observed in patients belonging to the higher staged groups. These results reveal the possible existence of chemoresistant “recurrence seeds”, predominantly in the peritoneal cavity. Needless to say, it is difficult to remove these invisible metastases by surgery alone; therefore, we expect that chemotherapy has a sufficient ability to eliminate occult clones. Indeed, most CCC patients undergo postoperative chemotherapy to prevent future recurrence as much as possible. Because CCC is considered to be an aggressive malignancy, adjuvant chemotherapy has been recommended with a view that it may actually be effective treatment. However, the aforementioned results suggest that the advantage of postoperative chemotherapy may fall short of our expectations. Actually, in the current study, we did not identify any significant differences in the rates of recurrence or death between the chemotherapy-present and absent groups. Similarly, a previous report by Timmers et al. reveled that there was no difference in the recurrence-free survival of patients with early-stage CCC with or without chemotherapy, despite the fact that there was a significant difference in the response of patients with serous carcinoma [7]. In addition, based on a retrospective study examining 219 patients with stage I CCC, including 195 patients who received adjuvant chemotherapy and 24 patients who did not, there were no significant differences in progression-free or overall survival between the two groups [8]. With regard to the number of chemotherapy cycles, Prendergast et al. reported that analyzing 38 (18.1%) patients received 3 cycles, and 172 (81.9%) patients received 6 cycles of mainly carboplatin-paclitaxel chemotherapy, and recurrence was comparable between the groups (18.4 vs. 27.3% for 3 vs. 6 cycles, respectively, N.S.). There was no impact of 3 versus 6 cycles of chemotherapy on recurrence-free or overall survival on univariate analysis (*NS*) [9]. These observations demonstrated that the effect of postoperative chemotherapy in CCC patients is so limited that the control of the budding of “recurrence seeds” and subsequent proliferation is difficult. However, since these results were derived from retrospective studies, we cannot draw a definite conclusion at present. A sophisticated prospective study will be necessary to answer this question in the future.

Confining analysis to 134 patients who eventually experienced recurrence, more than half of those patients died of the disease within approximately 2 years after recurrence. Actually, recurrent CCC is thought to be an extremely aggressive tumor, showing resistance to salvage treatment. Through a search of 344 cycles in 51 patients with recurrent CCC, Crotzer et al. reported that among patients with even platinum-sensitive disease (n=22 regimens), 2 patients (9%) showed partial responses to retreatment with carboplatin plus paclitaxel, and 4 (18%) had stable disease. In addition, among patients with platinum-resistant disease (n=83 regimens), only 1 patient (1%) showed a partial response [10]. In contrast, to date, a variety of lines of evidence regarding therapeutic modalities other than chemotherapy have been reported, including adjuvant radiotherapy or salvage immunotherapy. Several clinical series have also demonstrated that adjuvant radiotherapy may be of greater value in CCC than EOC of other histological types. Nagai et al. reported 16 stage IC-III CCC patients with postoperative whole abdominal radiotherapy, compared those with chemotherapy alone. The 5-year overall and disease-free survival rates in the radiotherapy group were 81.8 and 81.2%, respectively, which were significantly higher than those in the chemotherapy group (33.3 and 25.0%, respectively) [11]. In contrast, the 5-year OS and DFS rates in the CAP group were 33.3 and 25.0%, respectively. However, in the subset analysis including 59 patients with IC2/IC3 and stage II tumors from Hogen et al., adjuvant RT was not significantly correlated with a longer progression-free or overall survival, and the authors concluded that adjuvant radiotherapy was not associated with a survival benefit in these patients [12]. In addition, a review by Hoskins et al. (20) examined 241 patients with CCC treated with surgery followed by platinum-based chemotherapy or chemotherapy and abdominal radiotherapy. They identified no significant difference in 5-year disease-free survival between patients with stages IA and IC (with capsule rupture) with the addition of radiotherapy. Although the efficacy of adjuvant radiotherapy has yet to be clarified, these findings prompted us to hypothesize that radiotherapy is a therapeutic option for the treatment of CCC patients. Furthermore, immunotherapy is expected to become an alternative therapeutic modality for CCC. To date, several studies explored the roles of PD-1 and PD-L1 as therapeutic targets in patients with ovarian cancer, including CCC [13, 14]. In addition, Suzuki, et al. reported that immunotherapy based on glypican-3 peptide vaccinations has the potential to prolong the survival of patients with refractory CCC, allowing them to maintain their quality of life with no serious toxicities [15]. Studies of biomarkers to select appropriate candidates for CCC patients and provision to minimize immune-related toxicities are needed for personalization of the treatment approach to this tumor. Given that CCC patients with more than stage IC2/IC3 tumor showed markedly poor oncologic outcome despite chemotherapy, we are hopeful for positive results from large-scale prospective studies on these additional treatments.

The current study is inconclusive because of its retrospective nature and patient accumulation from multiple institutions over a long time. In addition, we were unable to evaluate explicit information about salvage chemotherapy and secondary cytoreductive surgery. Furthermore, one of the major limitations of the current study was that not all of the patients underwent systematic lymphadenectomy. In such patients, we may have missed the nodal occult metastases. In contrast, the strength of our study includes the central pathologic review, resulting in less intraobserver variability on determining the histological type. Additionally, the initial surgery and treatment were carried out based on generally similar treatment strategies. In the current examination, all patients underwent peritoneal staging, including ascites/washing cytology and information on the capsule state. Furthermore, adjuvant chemotherapy regimens were also well-defined by the original study protocols based on the standard treatment.

In conclusion, to our knowledge, this is one of the largest studies on oncologic outcomes in CCC patients who achieved complete tumor resection. Our data reiterate the aggressive chacteristics of the IC2/IC3 tumor. To date, the treatment strategy for CCC has been basically the same as those for other histologic types of EOC. Further clinical trials, aiming at individual treatment for CCC, are necessary, thereby shedding light on the optimal strategy to treat this tumor.

## MATERIALS AND METHODS

### Patient enrollment

**Figure 6:**
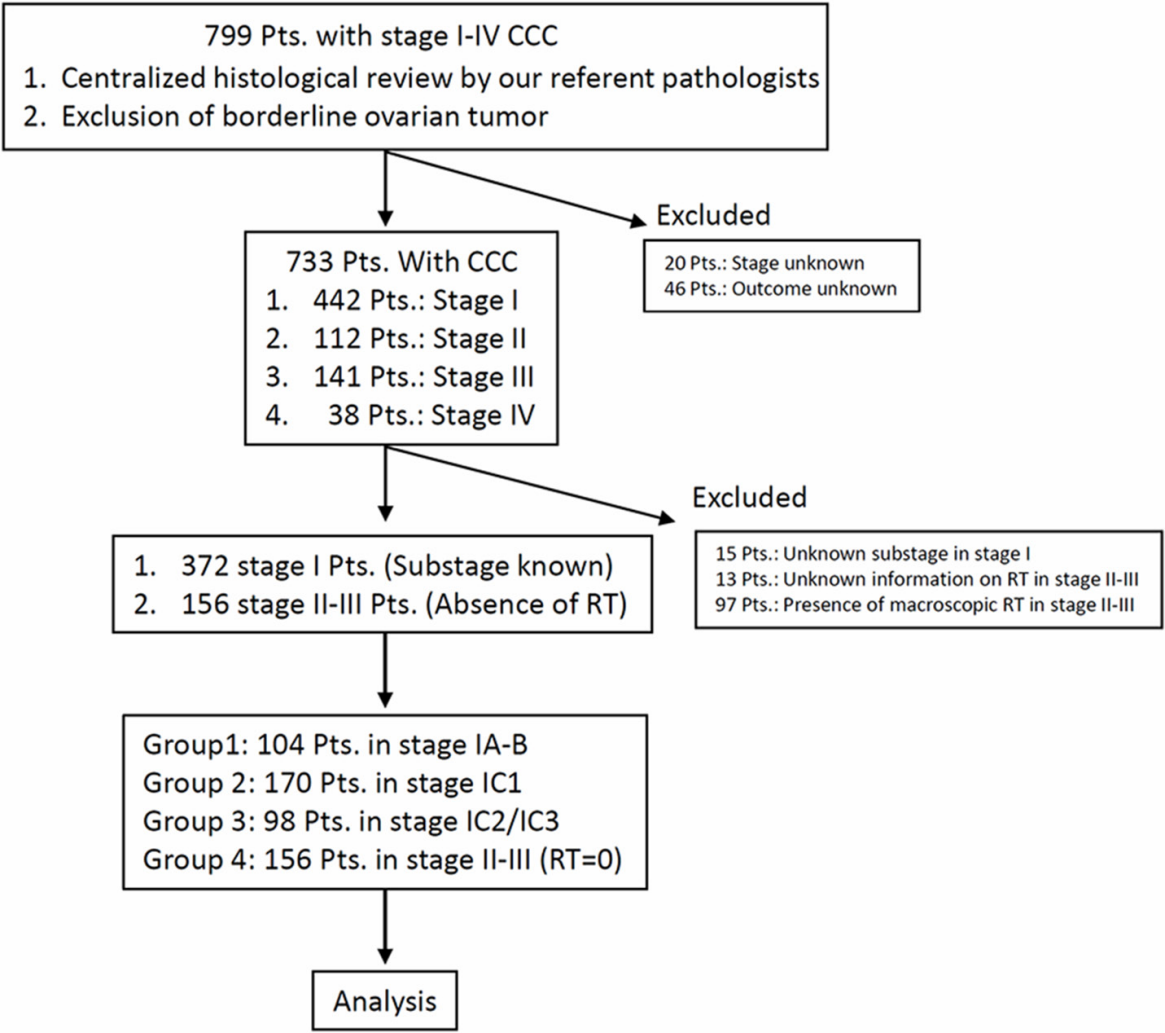
Flowchart of patient inclusion

Patients with malignant ovarian tumors have been registered and accumulated by the Tokai Ovarian Tumor Study Group (TOTSG), consisting of 14 collaborating institutions; Nagoya University Hospital, Aichi Cancer Center Hospital, Anjyo Kosei Hospital, Toyohashi Municipal Hospital, Toyota Memorial Hospital, Ogaki Municipal Hospital, Nagoya First Red-cross Hospital, Nagoya Second Red-cross Hospital, Nagoya Ekisaikai Hospital, Nagoya Memorial Hospital, Okazaki Municipal Hospital, Handa City Hospital, Komaki City Hospital, and Gifu Prefectural Tajimi Hospital. All histological slides were reviewed by two expert pathologists with no knowledge of the patients’ clinical data under a central pathological review system, but they did have minimal information oo the macroscopic tumor status. Between 1990 and 2015, 799 patients with ovarian CCC were identified in this registry system. Eligible cases included: (1) patients who received initial surgery and periodic follow-up at the aforementioned institutions; (2) patients for whom there was sufficient information on the residual tumor at primary surgery, first-line chemotherapy, and date of recurrence or death; and (3) patients diagnosed with CCC due to typical clear or hobnail cells growing in a papillary, solid, or tubulocystic pattern based on a central pathological review system (the criteria of the World Health Organization). Sixty-six patients were excluded from this study due to insufficient clinical data, a history of other malignancies, or being lost to follow-up immediately after surgery, leaving 733 CCC. A further125 patients were excluded from this study due to: 1) missing information on RT in stage II-III (N=15), 2) missing information on RT in stage II-III (N=13), and 3) the presence of macroscopic RT in stage II-III (N=97). Therefore, 528 patients with CCC were finally enrolled (Figure 6). The stage was defined according to the classification of the International Federation of Gynecology and Obstetrics (FIGO, 1988). In addition, substage of stage IC were categorized into three subtypes based on the FIGO (2014) classification [16]. For convenience, we classified patients into four groups: Group 1: FIGO stage IA-IB (N=104), Group 2: FIGO stage IC1 (N=170), Group 3: FIGO stage IC2/IC3 (N=98), and Group 4: FIGO stage II-III (no residual tumor: N=75). This study was approved by the ethics committees of Nagoya University.

### Treatment

Primary laparotomy was conducted in all patients to facilitate assessment of the abdominal contents. In principle, standard primary surgical treatment consisted of hysterectomy, bilateral salpingo-oophorectomy, infracolic omentectomy, retroperitoneal lymphadenectomy, or sampling. Nineteen patients underwent conservative surgery because they hoped to preserve fertility and were young. In those patients, at least, unilateral

salpingo-oophorectomy with peritoneal staging was performed. Peritoneal washing was routinely carried out in all patients. If any abnormalities were identified, peritoneal biopsies from different sites were appropriately performed. If patients were at an advanced stage, or showed severe perioperative complications and/or comorbidity, or underwent conservative surgery, retroperitoneal lymphadenectomy was not performed at each surgeon’s discretion.

In all, approximately 70% of patients underwent simple hysterectomy, bilateral salpingo-oophorectomy, omentectomy, and full surgical staging including pelvic and paraaortic lymphadenectomy/sampling. Detailed distributions of the surgical procedure in each group are shown in Supplementary Table 1. As patients with CCC showed poorer clinical outcomes, chemotherapy was in principle recommended for all patients; however, in 68 women, this was not done. Policies regarding chemotherapeutic agents varied over time; however, we basically used the same selection criteria for first-line regimens as TOTSG. Details of the chemotherapy regimens during each period were described previously [17].

### Follow-up and analysis

At the end of treatment, all patients underwent a strict follow-up, consisting of clinical checkups such as a pelvic examination, ultrasonography scan, CA125 evaluation, and periodic radiologic image. Radiologic recurrence was defined as tumor recurrence based on computed tomography (CT), magnetic resonance imaging (MRI), PET (positron emission tomography), and/or ultrasound, and clinical recurrence was defined as the development of ascites, elevated CA125, or a clinically palpable mass according to the Gynecologic Cancer InterGroup (GCIG) criteria in principle [18]. Recurrence-free survival was defined as the time interval between the date of initial surgery and that of recurrence, death, or the last follow-up, and cumulative incidence curves were fitted (CIR: Cumulative incidence of recurrence). Cancer-specific survival was defined as the time interval between the date of initial surgery and that of cancer-specific death or the last follow-up, and cumulative incidence curves were fitted (CID: Cumulative incidence of cancer-specific death). Postrecurrence cancer-specific survival was defined as the time interval between the date of recurrence and that of cancer-related death or the last follow-up. Survival curves were based on the Kaplan–Meier method. The survival curves were compared employing the Log-rank test. A *P*-value of < 0.05 was considered significant.

## SUPPLEMENTARY MATERIALS FIGURE AND TABLE



## Abbreviations

CCC: clear-cell carcinoma
RT: residual tumors
CIR: cumulative incidence of recurrence
CID: cumulative incidence of death
PRS: post-recurrence survival
