# Supplementary material for: The possible existence of occult metastasis in patients with ovarian clear-cell carcinoma who underwent complete resection without any residual tumours
Source: Oncotarget. 2018 Jan 4;9(5):6298–307. doi: 10.18632/oncotarget.23921 (PMC5814213; doi:10.18632/oncotarget.23921)
Supplement: Supplementary file 1 [file oncotarget-09-6298-s001.pdf]

The possible existence of occult metastasis in patients with ovarian clear-cell carcinoma who underwent complete resection without any residual tumours

SUPPLEMENTARY MATERIALS

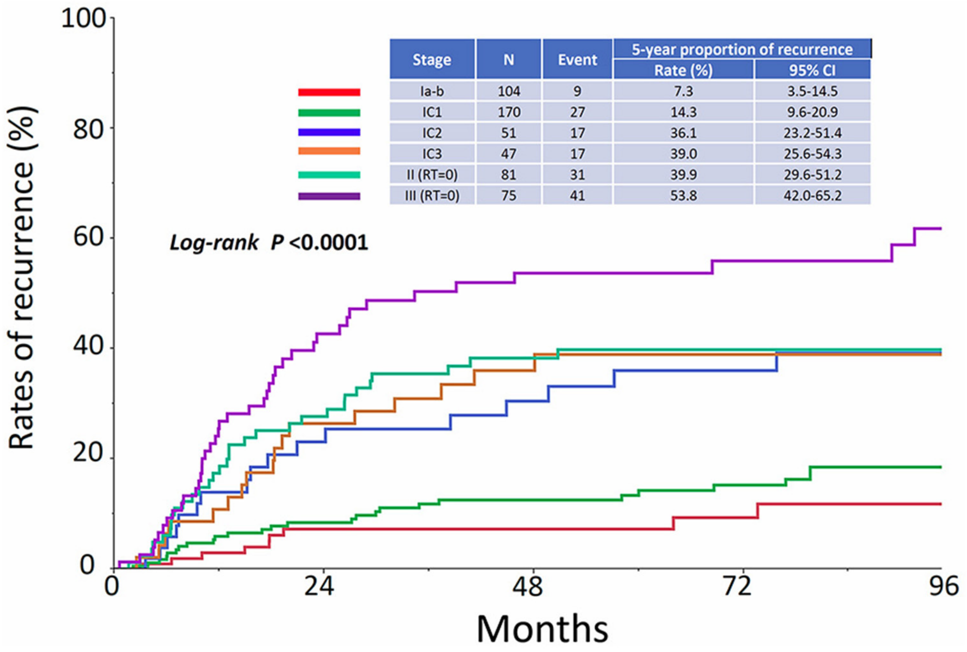

Supplementary Figure 1: The CIR of CCC patients belonging to each stage.

Supplementary Table 1: Distributions of the surgical procedure in each group

	Total		Group 1		Group 2		Group 3		Group 4	
	N	%	N	%	N	%	N	%	N	%
STH+BSO+OM+full staging[#1]	354	67.0	64	61.5	112	65.9	65	66.3	113	72.4
STH+BSO±OM	143	27.1	33	31.7	44	25.9	28	28.6	38	24.4
USO/BSO±OM	12	2.3	2	1.9	4	2.4	3	3.1	3	1.9
FSS	19	3.6	5	4.8	10	5.9	2	2.0	2	1.3
Total	528	100.0	104	100.0	170	100.0	98	100.0	156	100.0

STH: simple hysterectomy, BSO: bilateral salpingo-oophorectomy, OM: omentectomy, USO: unilateral bilateral salpingo-oophorectomy, FSS: fertility-sparing, surgery, #1: including pelvic and paraaortic lymphadenectomy/sampling. Each surgery including intestinal resection, if necessaly.
